# Porous Titanium Scaffolds Fabricated by Metal Injection Moulding for Biomedical Applications

**DOI:** 10.3390/ma11091573

**Published:** 2018-09-01

**Authors:** Ali Dehghan-Manshadi, Yunhui Chen, Zhiming Shi, Michael Bermingham, David StJohn, Matthew Dargusch, Ma Qian

**Affiliations:** 1 Queensland Centre for Advanced Materials Processing and Manufacturing (AMPAM), School of Mechanical and Mining Engineering, The University of Queensland, St Lucia, QLD 4072, Australia; zmshi@uq.edu.au (Z.S.); m.bermingham@uq.edu.au (M.B.); d.stjohn@uq.edu.au (D.S.); m.dargusch@uq.edu.au (M.D.); 2 Department of Mechanical Engineering, University College London, Torrington Place, London WC1E 7JE, UK; yunhui.chen@ucl.ac.uk; 3 School of Engineering, Centre for Additive Manufacturing, RMIT University, Melbourne, VIC 3000, Australia; ma.qian@rmit.edu.au

**Keywords:** titanium, metal injection moulding, space holder, scaffold, mechanical properties, corrosion

## Abstract

Biocompatible titanium scaffolds with up to 40% interconnected porosity were manufactured through the metal injection moulding process and the space holder technique. The mechanical properties of the manufactured scaffold showed a high level of compatibility with those of the cortical human bone. Sintering at 1250 °C produced scaffolds with 36% porosity and more than 90% interconnected pores, a compressive yield stress of 220 MPa and a Young’s modulus of 7.80 GPa, all suitable for bone tissue engineering. Increasing the sintering temperature to 1300 °C increased the Young’s modulus to 22.0 GPa due to reduced porosity, while reducing the sintering temperature to 1150 °C lowered the yield stress to 120 MPa, indicative of insufficient sintering. Electrochemical studies revealed that samples sintered at 1150 °C have a higher corrosion rate compared with those at a sintering temperature of 1250 °C. Overall, it was concluded that sintering at 1250 °C yielded the most desirable results.

## 1. Introduction

Titanium (Ti) and its alloys are advanced metallic materials with a range of unique properties such as a high strength to density ratio, superior corrosion resistance and excellent biocompatibility that make them desirable materials for the manufacture of hard tissue components [1,2,3]. Orthopaedic and dental implants for load-bearing applications are successful examples in that regard [4,5,6,7]. However, there are concerns regarding the long-term performance of titanium implants due to their mismatch in mechanical properties (especially the Young’s modulus) with those of natural bone, which can lead to the stress shielding effect [8]. Any attempt to modify the mechanical properties of titanium for an improved match is therefore desired. The development of new titanium alloys, new manufacturing techniques and porous structures are among the successful efforts to make titanium alloys more bone-like [9,10,11,12,13,14,15].

Introducing a sufficient amount of porosity has proven to be effective in tailoring the modulus of a solid material. Additionally, surface porosity with a pore size in a certain range has proved to be able to enhance cell responses. Traditionally, porous polymers and ceramic materials have been used as synthetic bone craft scaffolds for a number of years [16,17]. However, the mechanical properties of these materials are usually insufficient for load-bearing applications, such as stand-alone interbody spinal fusion devices [10]. In that regard, porous metals are better options.

As a unique powder metallurgy (PM) approach, metal injection moulding (MIM) is capable of producing both porous and dense small titanium (and other metal) components with significant design flexibility [18,19,20,21]. MIM combines the attributes of PM (e.g., low cost, simplicity, and flexibility of composition selection) with those of plastic injection moulding (e.g., the ability to manufacture complex parts and rapid production) [19]. This combination has enabled the MIM process to be used as an attractive and economic manufacturing technique for a variety of medical and dental components [22,23,24]. However, to date, MIM has been mainly used to manufacture dense components. If highly porous structures are desired, special MIM techniques need to be developed. Dispersing temporary space holders into MIM feedstock is one potential solution to this challenge. A US patent filed in 2003 by Nelles et al. [25] is among the first attempts to provide a MIM-based manufacturing technique for porous metals including titanium using space holders (e.g., KCl or NaCl). Since then, the process has encouraged follow-up research activities. Although there is no industrial production currently of porous medical implants using MIM technology, there is much ongoing research and development effort to evaluate and develop the technique [26,27,28,29,30,31,32,33,34,35,36]. For example, Carreno-Morelli et al. [26] manufactured Ti parts with a porosity of up to 60% using MIM of titanium hydride powder and the space holder technique with modulus (4–22 GPA) similar to that of human bone. Chen et al. [28] fabricated porous Ti parts with up to 60% porosity using hydride–dehydride (HDH) Ti powder and NaCl as the space holder. However, the optimum parameters for the MIM manufacturing of porous Ti parts with the desired interconnected porosity, mechanical properties and dimensional accuracy are yet to be established.

This study aims to identify the suitable MIM process parameters that are able to produce porous Ti parts with controlled porosity and mechanical properties. In this regard, samples were fabricated by MIM of Ti powder using potassium chloride (KCl) as the space holder. The resulting shrinkage, density, pore interconnectivity, mechanical and corrosion properties were characterised over a range of sintering temperatures.

## 2. Materials and Methods

### 2.1. Feedstock Preparation

**Figure 1:**
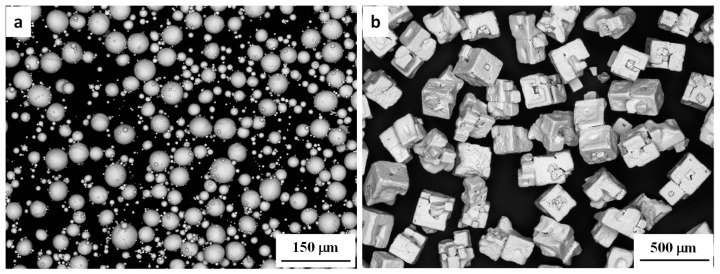
SEM image of (**a**) initial titanium powder and (**b**) potassium chloride (KCl) powder.

Spherical gas-atomised commercially pure Ti powder of >99.6% purity and a particle size of <45 µm (Advanced Powders and Coatings Inc., Quebec, QC, Canada) and cuboidal potassium chloride (KCl) with a particle size of <250 µm (Sigma Aldrich, St. Louis, MO, USA) were used as the starting powder and space holder, respectively, as shown in Figure 1. The manufacturer report shows that Ti powder has more than 95% particles smaller than 45 µm. A previously-assessed [37] simple binder system was selected, which consisted of 61 wt % paraffin wax (PW) from Sigma-Aldrich, 36 wt % high density polyethylene (HDPE) from Qenos, Melbourne, Australia and 3 wt % stearic acid (SA) from Sigma-Aldrich. For feedstock preparation, a solid loading of 69% (i.e., the volume ratio of Ti + KCl to the binder) was selected for smooth injection moulding. Previous works have shown that this solid loading performs well during mixing and injection moulding [21]. Appropriate proportions of Ti powder, KCl (40 vol %) and binder were dry mixed for 60 min in a Turbula 3D mixer. The dry mixture was then loaded into a pre-heated laboratory scale sigma mixer and mixed at 150 °C for 2 h under an argon atmosphere. The mixture was subsequently unloaded from the mixer and cooled to room temperature. To make the mixture even more homogenous, it was loaded into a EuroLab 16 twin screw extruder (ThermoFisher Scientific, Karlsruhe, Germany) that was preheated to 160 °C and extruded several times to produce a uniform mixture. After that, the mixture was cooled to room temperature and hand-crushed into granules of <3.0 mm in size as the feedstock for the MIM process.

### 2.2. Injection Moulding, Debinding and Sintering

**Figure 2:**
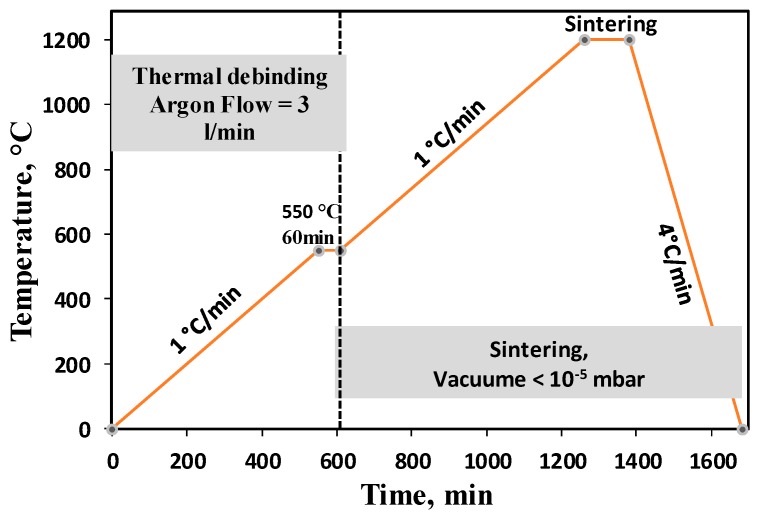
The schematic diagram of debinding and sintering processes for porous scaffolds.

The prepared feedstock was injection moulded into Φ 12 mm × 22 mm cylindrical samples using a micro-injection moulding machine (Babyplast 610P, Barcelona, Spain). After moulding, samples were immersed in a hexane bath at 50 °C for 20 h to remove the paraffin wax from the binder. Our previous study [37] confirmed that a complete removal of the paraffin wax requires 20 h of immersion at 50 °C. After solvent debinding, samples were immersed in heated water (60 °C) for 24 h to extract the space holder (KCl). To evaluate thermal debinding parameters, thermogravimetric (TGA) measurements on HDPE were performed using a Netzsch STA409 instrument (Netzsch, Selb, Germany) under a protective argon gas atmosphere and different heating rates. The thermal de-binding of MIM samples was performed by slow heating of samples to 550 °C in argon at a flow rate of 3 L/min for an isothermal hold of 1.0 h. Sintering was carried out subsequently by switching the furnace to a high vacuum of <10^−5^ mbar. Samples were sintered at 1150 °C, 1250 °C or 1300 °C. Figure 2 illustrates the thermal de-binding and sintering details.

### 2.3. Materials Characterisation

The as-sintered density was measured using the Archimedes method (the theoretical density of Ti was taken as 4.506 g/cm^3^) and H-Galden ZT-180 fluid (Solvey, Milan, Italy). The open porosity was calculated as

(1) $$\;P_{Open\;}=\frac{\rho_{HG}\left(M_{Oil}-M_{Air}\right)}{\rho_{Oil}\left(M_{Oil}-M_{HG}\right)}\times 100$$

where *ρ_HG_* is the density of the H-Galden (1.697 g/mL at 23 °C), *ρ_Oil_* is the density of oil (KS7470, density 0.885 g/mL), *M_Air_* is the dry mass of the sintered sample, *M_Oil_* is the mass of the sample after oil infiltration, and *M_HG_* is the mass of the oil infiltrated sample measured while immersed in H-Galden. The pore interconnectivity was assessed from the ratio of open porosity to the total porosity (overall porosity). Also, the 3D size, shape and distribution of pores were assessed using computed tomography (Micro-CT). In this regard, the cross-sections of the samples were recorded in three directions with step sizes of 0.01 mm. Then, reconstruction software (Mimics^®^ 19.0, Materialise, Leuwen, Belgium) was used to create the 3D model of each porous structure.

Compression properties were measured with an Instron 5584 machine (Instron Inc., High Wycombe, UK) at a cross-head movement rate of 0.01 mm/min. The 0.2% yield strength (σ_0.2_), strength at 40% deformation (σ_40_) and Young’s modulus were obtained.

Samples for microstructural characterisation and pore analysis were cut longitudinally along the cylindrical axis followed by standard metallographic preparation. A Hitachi TM3030 scanning electron microscope (SEM) (Hitachi, Tokyo, Japan) was used for characterisation of powder and pore structures.

### 2.4. Corrosion Testing

In order to evaluate the effect of the porosity and sintering temperature of the MIM sample on the corrosion behaviours of the porous titanium samples, corrosion tests were performed on Ti porous samples sintered at 1250 °C (Ti-1250) and 1150 °C (Ti-1150) in a simulated body corrosive environment. The electrodes were prepared by mounting porous titanium in epoxy cold resin and were cured for 12 h, leaving the surface area of 0.83 cm^2^ and 0.65 cm^2^ for Ti-1250 and Ti-1150 alloys, respectively. The surface exposed to Hanks solution was gradually ground using SiC paper from 320 grit to 1200 grit, followed by rinsing with ethanol as well as ultrasonic cleaning in ethanol for 5 min. The open circuit potential, Ecorr, was measured immediately after the sample was immersed in Hank’s balanced salt solution. The samples were immersed in Hank’s solution for 2 h with open aeration at 37.5 °C in the temperature controlled water bath. The open circuit potential was measured until it became stable. The electrochemical impedance spectra (EIS) was measured after 2 h of immersion followed by measurement of the polarisation curve. Hank’s balanced salt solution was prepared by mixing one prepacked SIGMA H1387-1L (Sigma, Steinheim, Germany) powder and 90% of final required volume of distilled water. The mixture was gently stirred until all powder was dissolved. The original package rinsed with a small amount of water to remove all traces of powder and then added to the above solution. Then, 0.35 g of sodium bicarbonate powder was added to the final volume of solution and stirred until completely dissolved. Finally, additional water was added to bring the solution to 1.0 L. The composition of Hank’s balanced salt is listed in Table 1.

A three-electrode cell arrangement was utilised for the electrochemical measurements, with a silver/silver chloride saturated with potassium chloride (Ag/AgCl Sat. KCl) as the reference electrode and a platinum foil as the counter electrode. All potential values in this work are versus the Ag/AgCl electrode saturated with KCl. The polarisation and EIS tests were repeated to evaluate the reproducibility of the results. EIS was carried out in the frequency range from 100 KHz to 10 MHz with AC amplitude of 10 mV at the corrosion potential using a Princeton Applied Research PARSTAT 2273 Advanced Electrochemical System controlled by Powersuite software 2.53 (Princeton Applied Research, Oak Ridge, TN, USA). After the EIS measurement, the polarisation curves was measured from −350 mV to 2.5 V versus open circuit potential by a potentiodynamic polarisation scan at the scanning rate of 0.166 mV/s. Experiments were conducted in naturally aerated Hank’s solution with 600 mL cell at 37.5 °C controlled by a thermostatic water bath.

## 3. Results and Discussion

### 3.1. Binder Assessment and De-Binding

**Figure 3:**
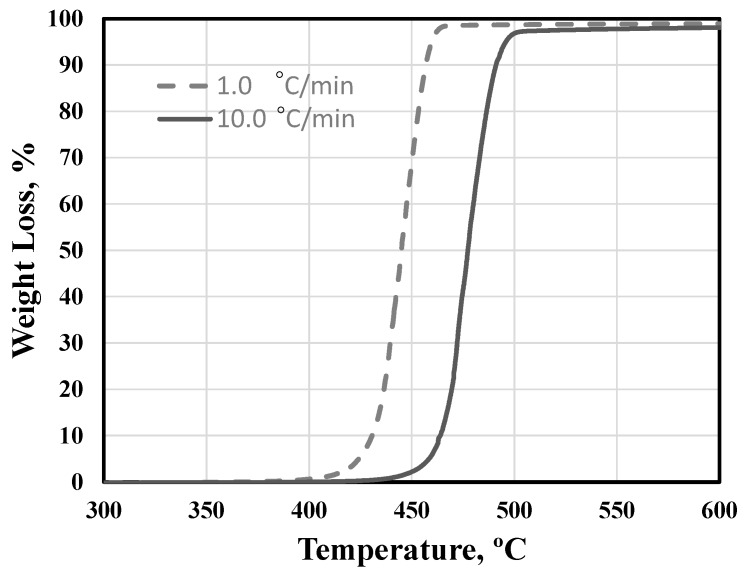
Thermal decomposition of high density polyethylene (HDPE) under two different heating rates.

The first step to evaluate the performance of a binder system during MIM is to understand the thermal response of the main polymer component. In that regard, thermogravimetric analyses (TGA) were performed on HDPE at different heating rates to identify its decomposition characteristics during heating. Figure 3 shows the TGA experiments carried out at heating rates of 1.0 °C/min and 10 °C/min. A fast decomposition rate of the HDPE was observed over the temperature range 420–470 °C at 1.0 °C/min and 470–500 °C at 10 °C/min. Thermal de-binding was therefore performed slowly over respective temperature ranges at selected heating rates in order to prevent the excessive distortion or even disintegration of samples (Figure 2). Our previous studies confirmed the suitability of this heating profile for complete thermal debinding of samples [37].

### 3.2. Shrinkage, Porosity and Pore Size Distribution

Dimensional shrinkage in both cylinder height (longitudinal) and diameter (radial) were calculated by measuring the sample dimensions before and after sintering. The results are presented in Table 2, which clearly revealed the significant effect of sintering temperature on shrinkage. However, this shrinkage is very consistent in both longitudinal and radial directions, indicating a well homogenised feedstock. The results of measured density and porosity fraction are included in Table 2. After sintering at 1150 °C, the final porosity of 42.5% is very close to the designed porosity of 40%. However, as expected, increasing the sintering temperature reduced the porosity of samples (i.e., 36% and 34% after sintering at 1250 °C and 1300 °C, respectively). The effect of sintering temperature on the final porosity and shrinkage could be related to the densification behavior of solid powders during sintering process. During the sintering process, individual powders bond together through the solid-state diffusion process. This process causes necking at the point of contact between adjacent particles as well as the boundary of the particles [38]. Increasing the sintering temperature can accelerate this necking process due to the increase in the solid-state diffusion rate, leaving less porosity between individual powders. However, there is also the gravitational force acting on the component during sintering. As the sintering temperature increases, the material is softer and the gravitational force can accelerate the material’s movement towards large internal pores (from space holder removal) and leave less porosity in components sintered at higher temperature (as seen in Table 2).

The density measurement data in Table 2 indicates that samples sintered at 1150 °C, 1250 °C and 1300 °C achieved densities of 2.60, 2.86 and 2.96 g/cm^3^, respectively. Healthy human bone mineral density (BMD) is around 3.88 g/cm^2^ for males and 2.90 g/cm^2^ for females [39]. The density ranges of the porous samples produced in this study are consistent with the BMD range, which can improve patient comfort and lower implant failure rate [12]. The results in Table 2 also showed that more than 90% of the pores are interconnected, which is essential for an implanted scaffold to allow body fluid transport and cell ingrowth [40].

**Figure 4:**
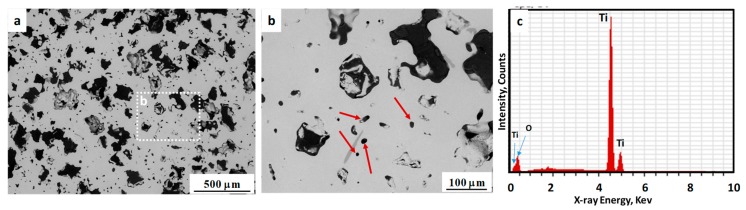
(**a,b**) SEM micrograph of porous Ti sintered at 1250 °C. Arrows show the micron size pores; (**c**) EDS analysis of scaffolds.

To better evaluate the pore structure and distribution in the as-sintered samples, the microstructures were examined using SEM. Figure 4 shows the SEM micrographs of polished cross-sections of the samples sintered at 1250 °C, revealing pore sizes, morphology and distribution (images taken from the center of samples cross section). In these images, some large and irregularly shaped pores of 150–200 µm resulting from space holder removal (Figure 4a), as well as micron-sized pores (Figure 4b) which formed due to the binder removal and sintering of Ti particles, are visible. Research has suggested that the optimum pore size for body fluid transport and mineralised bone ingrowth is in the range of 100–300 µm [40,41]. As seen in Figure 4b, the internal walls of those large pores have a rough surface, which can make them more effective for the ingrowth of new bone tissue [28]. Also, Figure 4c shows the energy-dispersive X-ray spectroscopy (EDS) of sintered samples, confirm no presence of Cl or K in the microstructure, indicative of thorough space holder removal during water immersion.

**Figure 5:**
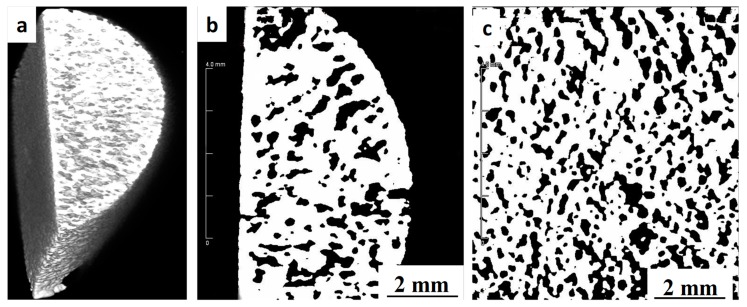
Computed micro-tomography image of the MIM proceed porous titanium sample sintered at 1250 °C: (**a**) 3D view of the sample, (**b**) side view and (**c**) top view.

The external and internal pore structures as well as the 2D surface and 3D space parameters, such as pore distribution and porosity related to bone histo-morphometry, were assessed using an Inveon multimodality preclinical PET/CT scanner (Siemens, Berlin, Germany) studies. Figure 5 shows the Micro-CT images of a sample sintered at 1250 °C, which is indicative of a high level of porosity interconnectivity and a uniform distribution inside the sample. Using ImageJ 1.8 software (National Institutes of Health, Washington D.C. USA) the porosity was measured to be approximately 36%, which is very close to the results obtained from the Archimedes method. The open pores extend from the surface through to the center of the scaffold which is beneficial for osseointegration as they can facilitates the transport of nutrients and oxygen required for vascularization during bone tissue development [12].

### 3.3. Mechanical Properties

**Figure 6:**
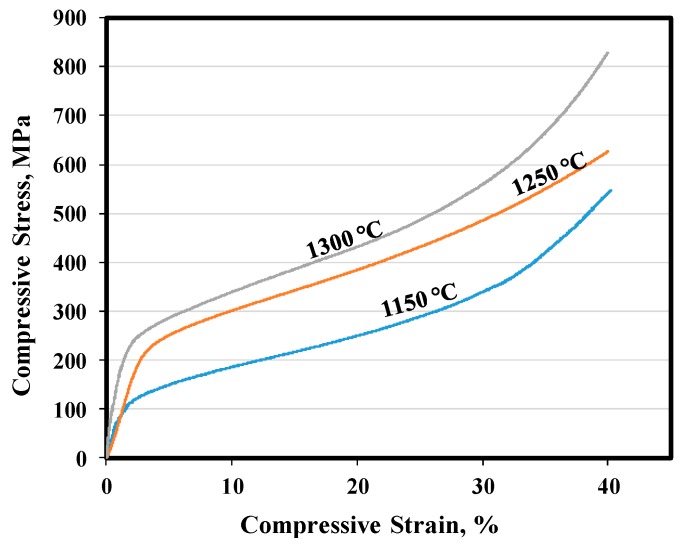
Compressive stress–strain curves of sintered samples at different temperatures.

Figure 6 shows the representative compression testing curves obtained from samples sintered at different temperatures. Also, the compressive strength at 40% compression (σ_40_), 0.2% yield strengths (σ_0.2_) and Young’s modulus are summarized in Table 3. In this table, similar properties for a cortical human bone have also been listed for comparison [42]. Both values of σ_0.2_ and σ _40_ were increased by increasing the sintering temperature, due to improved sintering. The Young’s modulus for all samples is very low and is comparable with that of cortical human bone [42]. Such an increase in the strength and modulus of samples by increasing sintering temperature could be related to an improvement in the bonding between individual Ti powders at higher temperatures, the level of densification (as seen in Table 2 by reducing porosity), grain coarsening and alterations in the microstructural morphology [43,44]. However, it is encouraging that the compressive properties fall well within the range of those for human natural bone. These promising results indicate the good mechanical suitability of the porous Ti samples manufactured by MIM for implant applications [45].

### 3.4. Evaluation of Corrosion Behaviours in Hank’s Solution

**Figure 7:**
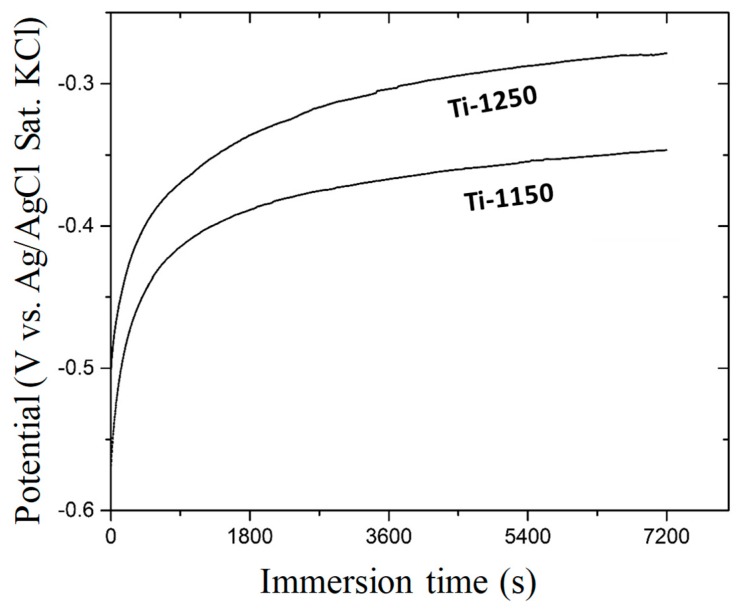
Open circuit potential vs immersion time of porous Ti samples sintered at 1250 °C (Ti-1250) and 1150 °C (Ti-1150) measured in Hank’s balanced salt solution at 37.5 °C for two hours.

Figure 7 shows the change in open circuit potential with immersion time in Hank’s solution at 37.5 °C. The initial potential for Ti-1250 was about −0.459 V (Ag/AgCl Sat. KCl). This potential increased rapidly in the beginning, followed by a gradual increase towards nobler potentials, and stabilised at −0.303 V after 2 h. For the Ti-1150 sample which had more porosity and pore interconnectivity (Table 2), the initial potential was about −0.560 V, which gradually increased afterwards and became stabilised at −0.347 V, similar to that after 2 h immersion. The increase in potential can be attributed to the passivation of the titanium sample in the solution, where the potential would reach a stable stage once the passive film was able to completely block the corrosion process. The higher open circuit potential of the sample Ti-1250, compared with that of Ti-1150, indicates that a more stable or denser passive film has formed on the surface of the sample Ti-1250 due to its lower porosity and pore interconnectivity. Also, the higher sintering temperature in the Ti-1250 sample created more inter-particle bonding between individual Ti powders, resulting in a more stable structure in the corrosive environment.

**Figure 8:**
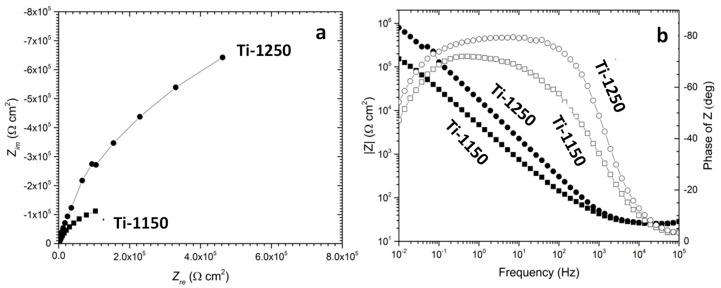
Electrochemical impedance spectra (EIS) of porous Ti samples sintered at 1250 °C (Ti-1250) and 1150 °C (Ti-1150) measured in Hank’s balanced solution at 37.5 °C after 2 h immersion testing: (**a**) Nyquist plots, (**b**) Bode plots. Solid circles indicate of samples sintered at 1250 °C and open circles indicate of samples sintered at 1150 °C.

**Figure 9:**
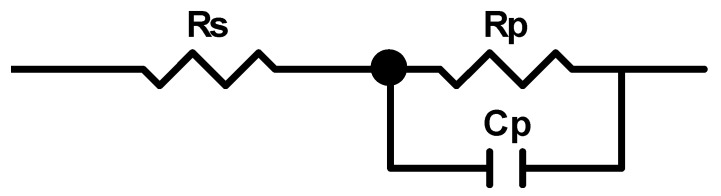
Equivalent circuit to simulate the corrosion of porous titanium alloys in Hanks’s balanced solution (*R_s_* corresponds to the resistance of solution, *R_p_* to the resistance of the oxide layer and *C_p_* to the capacitance of the barrier layer).

Figure 8 shows the EIS curves of the porous titanium immersed in Hank’s solution after 2 h at 37.5 °C in Nyquist and Bode plots. A typical semi-capacitive arc on the Nyquist plots was indicated from high frequency to low frequency at phase angles approaching −90°, suggesting that a highly stable film formed on both samples in Hank’s solution. This is consistent with the open circuit potential becoming nobler with increasing immersion time, as shown earlier in Figure 7. Based on the EIS features, the simple equivalent circuit shown in Figure 9 can be used to simulate the passivation process. The model assumes that the oxide layer formed on titanium alloys consists of a barrier-like layer only. In Figure 9, *R_s_* corresponds to the resistance of the solution, *R_p_* to the resistance of the oxide layer and *C_p_* to the capacitance of the barrier layer. A constant phase element was used as capacitance in simulating the process in order to simplify fitting.

**Figure 10:**
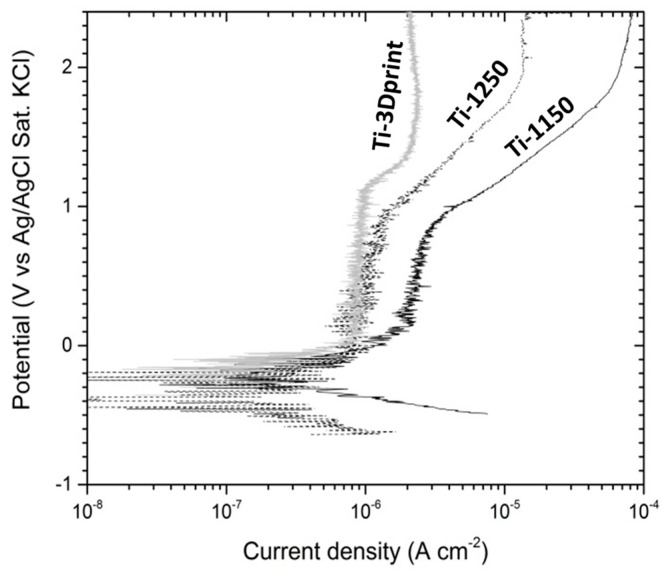
Polarisation curves of porous Ti samples sintered at 1250 °C (Ti-1250) and 1150 °C (Ti-1150) measured in Hanks balanced salt solution at 37.5 °C after two-hour immersion testing and EIS measurement (results from a dense Cp-Ti manufactured through 3D print reported as a comparison).

The dynamic polarisation curves of the two samples (i.e., Ti-1250 and Ti-1150) measured in Hank’s solution at 37.5 °C after 2 h immersion are shown in Figure 10. The average corrosion potential calculated from the polarisation curves are −0.297 V and −0.509 V for Ti-1250 and Ti-1150, respectively. Such a difference in the corrosion potential of samples sintered at different temperatures could be related to the effect of temperature on the microstructure and resulting porosity. As seen in Table 2, the porosity of the sample sintered at 1150 °C is about 6.0% more than the sample sintered at 1250 °C. Such increased porosity will increase the real surface of the sample which is in contact with the corrosive environment, resulting in a higher corrosion rate. Also, previous research [37] has shown that a sintering temperature of 1150 °C is not sufficient for MIM of Ti powder, resulting in incomplete sintering and, therefore, individual Ti powders cannot bond very well together. Such incomplete sintering can cause the partial disintegration of materials in a corrosive environment and higher corrosion rate. The calculated corrosion potentials are significantly lower than those obtained from the open circuit potential measurement, which could be due to oxide film removal during cathodic polarisation from −350 mV to the corrosion potential. The corrosion current density was worked out from the extrapolation of the cathodic polarisation curve to the corrosion potential. The low corrosion current density obtained from polarisation curves is ascribed to the self-passivation of titanium [46,47], and the difference in corrosion current density between two samples is related to the porosity in each sample. The test values are listed in Table 4.

The passivation current density (*I_p_*) increased slowly with a small fluctuation in the potential range from the corrosion potential to the film breakdown potential (E_b_), as shown in Figure 10. A 3D-printed dense commercially pure titanium sample was assessed for comparison and the potential current density curve was superimposed on Figure 10. It was noted that the current density was less stable in the two porous titanium materials than in the dense sample. The fluctuation in current density may be caused by the unstable polarisation inside the pores, with a larger drop in *IR*. For example, the *I_p_* value of the sample Ti-1150 was higher than that of the sample Ti-1250 in Figure 10 due to the higher porosity of Ti-1150 (Table 2). The higher porosity and larger fraction of interconnected pores in the sample Ti-1150 may have caused the formation of a non-uniform oxide film and therefore decreased the stability of the barrier layer [47].

## 4. Conclusions

Ti scaffolds with a porosity of up to 40% have been produced using the metal injection moulding process, assisted with the use of a space holder. The following conclusions can be made.

(1) MIM is able to manufacture porous biomedical titanium scaffolds with controlled shrinkage, density, porosity and a highly interconnected pore structure;
(2) Uniform shrinkage of around 12.0% was observed in all dimensions of the scaffold samples after sintering at 1250 °C or 1300 °C;
(3) Samples sintered at 1250 °C for 120 min achieved mechanical properties that are very close to those of human cortical bone;
(4) The corrosion resistance of scaffold titanium samples sintered at 1250 °C and 1150 °C in Hank’s solution changed with porosity. The higher the porosity, the lower the corrosion resistance;
(5) Overall, sintering at 1250 °C for 120 min can be chosen as a desired sintering condition in terms of the resulting porosity level (40%), mechanical properties, dimensional control and corrosion resistance.

## Figures and Tables

**Table 1:** Composition of Sigma H1387 Hank’s balanced salt.

Components	g/L
CaCl_2_	0.1396
MgSO_4_ (anhydrous)	0.09767
KCl	0.4
KH_2_PO_4_ (anhydrous)	0.06
NaCl	8.0
Na_2_HPO_4_ (anhydrous)	0.04788
D-Glucose	1.0

**Table 2:** Shrinkage, density and porosity fraction of metal injection moulding (MIM) samples sintered at different temperatures.

Sintering Temperature (°C)	Radial Shrinkage (%)	Longitudinal Shrinkage (%)	Density (g/cm^3^)	Overall Porosity (%)	Open Porosity (%)	Pore Interconnectivity (%)
1150	9.54 ± 0.65	9.81 ± 0.47	2.60 ± 0.05	42.5	40.6	95.5
1250	12.38 ± 0.77	12.62 ± 0.71	2.86 ± 0.05	36.5	33.4	91.5
1300	13.04 ± 0.69	13.21 ± 0.65	2.96 ± 0.03	34.4	33.8	98.2

**Table 3:** Mechanical properties of samples sintered at different temperatures.

Sintering Temperature (°C)	σ_0.2_ (MPa)	σ_40_ (MPa)	Young’s Modulus (GPa)
1150	123	553	8.40
1250	220	630	7.82
1300	230	831	21.69
Human cortical bone [38]	104–121	-	4–30

**Table 4:** Fitting results of the polarisation curves of porous Ti-1250 and Ti-1150 samples tested in Hanks solution at 37.5 °C.

Sample	E_corr_ (V)	I_corr_ (µA cm^−2^)	B_c_ (mV)	E_b_ (V)	E_tp_ (V)
Ti-1150	−0.297 ± 0.009	0.32 ± 0.06	−164 ± 22	0.806 ± 0.032	1.545 ± 0.338
Ti-1250	−0.510 ± 0.021	0.19 ± 0.02	−109 ± 1	0.874 ± 0.071	1.893 ± 0.014

E_corr_: corrosion potential; I_corr_: corrosion current density; B_c_: cathodic Tafel slope; E_b_: film breakdown potential; E_tp_: transpassivity potential.
